# Prediction of First and Multiple Antiretroviral Therapy Interruptions in People Living With HIV: Comparative Survival Analysis Using Cox and Explainable Machine Learning Models

**DOI:** 10.2196/78964

**Published:** 2026-02-04

**Authors:** Donald Salami, Emily Koech, Janet M Turan, Kristen A Stafford, Lilly Muthoni Nyagah, Stephen Ohakanu, Anthony K Ngugi, Manhattan Charurat

**Affiliations:** 1 Center for International Health, Education and Biosecurity, Institute of Human Virology, School of Medicine, University of Maryland, 725 W Lombard St, Baltimore, MD, 21201, United States, 1 410-706-8614; 2 Center for International Health, Education, and Biosecurity, Kenya, Nairobi, Kenya; 3 Department of Health Policy and Organization, School of Public Health, The University of Alabama, Birmingham, AL, United States; 4 Department of Strategic Information, Ministry of Health’s National AIDS and STI Control Program Kenya, Nairobi, Kenya; 5 Department of Population Health, Aga Khan University Nairobi, Nairobi, Kenya

**Keywords:** antiretroviral therapy, explainable machine learning, machine learning, recursive partitioning, treatment interruptions.

## Abstract

**Background:**

The Cox proportional hazards (CPH) model is a common choice for analyzing time-to-treatment interruptions in patients on antiretroviral therapy (ART), valued for its straightforward interpretability and flexibility in handling time-dependent covariates. Machine learning (ML) models have increasingly been adapted for handling temporal data, with added advantages of handling complex, nonlinear relationships and large datasets, and providing clear practical interpretations.

**Objective:**

This study aims to compare the predictive performance of the traditional CPH model and ML models in predicting treatment interruptions among patients on ART, while also providing both global and individual-level explanations to support personalized, data-driven interventions for improving treatment retention.

**Methods:**

Using data from 621,115 patients who started ART between 2017 and 2023, in Kenya, we compared the performance of the CPH with the following ML models—gradient boosting machine, extreme gradient boosting, regularized generalized linear models (Ridge, Lasso, and Elastic-Net), and recursive partitioning—in predicting first and multiple treatment interruptions. Explainable surrogate technique (model-agnostic) was applied to interpret the best performing model’s predictions globally, using variable importance and partial dependence profiles, and at individual level, using breakdown additive, Shapley Additive Explanations, and ceteris paribus.

**Results:**

The recursive partitioning model achieved the best performance with a predictive concordance index score of 0.81 for first treatment interruptions and 0.89 for multiple interruptions, outperforming the CPH model, which scored 0.78 and 0.87 for the same scenarios, respectively. Recursive partitioning’s performance can be attributed to its ability to model nonlinear relationships and automatically detect complex interactions. The global model-agnostic explanations aligned closely with the interpretations offered by hazard ratios in the CPH model, while offering additional insights into the impact of specific features on the model’s predictions. The breakdown additive and Shapley Additive Explanations explainers demonstrated how different variables contribute to the predicted risk at the individual patient level. The ceteris paribus profiles further explored the time-varying model to illustrate how changes in a patient’s covariates over time could impact their predicted risk of treatment interruption.

**Conclusions:**

Our results highlight the superior predictive performance of ML models and their ability to provide patient-specific risk predictions and insights that can support targeted interventions to reduce treatment interruptions in ART care.

## Introduction

The Cox proportional hazards (CPH) model has long been a foundational tool for time-to-event analysis, widely used in health care research to examine treatment interruptions, including among patients on antiretroviral therapy (ART). The CPH model is a favorable choice due to its straightforward interpretability; its coefficients can be easily understood as hazard ratios, which quantify the relative effect of each covariate on the likelihood of an event, offering clear insights for clinical decision-making and risk assessment [1,2]. In addition, its flexibility in handling time-dependent covariates allows it to incorporate variables that change over time, providing a more accurate reflection of patient dynamics [3-5]. Our previous work [6] focused on using time-dependent covariates within the CPH model to enhance its application in modeling the complex patterns of multiple treatment interruptions in patients on ART, aiming to capture the nuanced and changing influences on treatment adherence.

Despite its popularity, the CPH model has limitations. A key disadvantage is its handling of high-dimensional data with many covariates, as it can be prone to overfitting and may yield unreliable results due to multicollinearity [4]. This risk is particularly pronounced in time-dependent CPH models, where changing covariates can amplify the potential for erroneous inferences [4]. In addition, as a semiparametric model, it does not fully specify the baseline hazard function, potentially limiting its ability to capture complex, nonlinear relationships [7]. These limitations highlight the need for more flexible modeling approaches, especially when working with complex health care datasets with nonlinear, time-varying, or high-dimensional characteristics.

Machine learning (ML) models are increasingly used in time-to-event analysis due to their ability to capture complex, nonlinear relationships at scale. In HIV and ART research, however, most survival ML applications such as random survival forests and gradient boosting primarily rely on baseline or time-invariant covariates, limiting their ability to reflect the dynamic nature of ART care. Although some methods, including recursive partitioning (RP), have been adapted to incorporate temporal structures and time-varying effects [8-10], support for fully time-varying covariates remains limited in many survival ML frameworks. RP provides a flexible alternative by modeling nonlinear effects and interactions with time-varying covariates, yet its application to large-scale, longitudinal ART cohorts has been limited. In addition, advances in model interpretability have made ML models more explainable, helping to bridge the gap between predictive accuracy and practical applicability. Through methods such as permutation importance, partial dependence plots, and Shapley Additive Explanations (SHAP) values [11-13], ML models now provide insights into variable importance and individual predictions, making them increasingly useful for clinical decision-making and patient-centered care.

The aim of this paper is first to compare the performance of traditional CPH model and ML models in predicting treatment interruptions among patients on ART. This comparison aims to evaluate whether ML models, with their flexibility in handling nonlinear relationships and time-dependent covariates, can provide more accurate and reliable predictions than the CPH model. Second, provide clear, intuitive explanations for the predictions at both the global level to understand overall patterns and key risk factors and the local level for individualized predictions for a patient. By combining predictive accuracy with interpretability, this study aims to support health care providers in identifying patients at high risk for treatment interruption and in implementing personalized interventions to enhance treatment retention.

## Methods

### Study Population and Data

The analysis used longitudinal, deidentified electronic medical record (EMR) data from patients initiated on ART between January 1, 2017, and November 30, 2023, across 2156 facilities in all 47 counties of Kenya. Eligible patients had at least 2 follow-up visits or drug pickups after ART initiation, were followed for more than 28 days postinitiation, and had returned to care if they had experienced an initial treatment interruption.

### Data Preprocessing and Definitions

The preprocessing, cleaning, feature engineering, and covariate definitions are detailed in our previous work [6]. Treatment interruption was defined as no ART drug pickup and no clinical contact for greater than 28 days after the last expected contact [14]. Time-invariant covariates included age, gender, education, marital and employment status, noncommunicable disease, baseline regimen, and ART initiation year, while time-varying covariates were adherence, alcohol intake, World Health Organization stage, viral load, clinical stability assessment, regimen line, multimonth dispensing (MMD), differentiated service delivery (DSD) model, and prevention with positives package. Full list of covariates and description is included in Table S1 in Multimedia Appendix 1. A key preprocessing step included creating an “unknown” category for categorical variables with missing data. Engineered features included the COVID-19 pandemic period—dummy variable to indicate the COVID-19 pandemic; missed appointment—failure to attend a scheduled visit within 1-3 days of the scheduled date; defaulted appointment—failure to attend a scheduled visit within 4-28 days; and the duration of treatment interruption—average duration of any treatment interruption before a patient returned to care.  

### Analysis and Models

We performed 2 sets of modeling, that is, time-invariant model, for first-ever treatment interruption, and a time-varying model, for multiple interruptions. The data structure differs between the models: in a time-invariant model, covariates were fixed, with 1 row per patient containing the time to interruption and interruption status. In a time-varying model, covariates change over time, with multiple rows per patient representing different time intervals and multiple interruption statuses.

Our base reference model was the traditional CPH model. The time-invariant CPH assessed the effect of fixed covariates on time to first interruption, with an assumption that the hazard ratio remains constant over time, while the time-varying allowed covariates to change over time, capturing the dynamic effects of these covariates on the hazard at different intervals.

Our choice of ML models was based on algorithms with the ability to fit time-varying covariates and their computational feasibility at scale. We evaluated 3 classes of algorithms: boosting methods for their efficiency and predictive performance, regularized shrinkage models for handling multicollinearity and improving generalization, and RP for its ability to capture nonlinear relationships and interactions while remaining interpretable. Although random survival forests are widely used in survival analysis, their computational demands and limited support for fully time-varying covariates constrained their applicability in this study. The trained models are as described:

- Gradient boosting machine (GBM): an ensemble learning method that builds a series of decision trees, where each new tree corrects the errors of the previous ones. It is powerful for time-invariant survival analysis due to its ability to handle complex, nonlinear relationships and improve predictive accuracy by minimizing loss iteratively.
- Extreme gradient boosting (XGBoost): an optimized version of GBM, known for its speed and performance, especially with large datasets. It is well suited for our analysis due to its efficiency, scalability, and ability to manage missing data, offering robust predictions in survival tasks. The Accelerated Failure Time model was trained for a time-varying dataset.
- Regularized generalized linear models: Extends traditional regression models with regularization techniques—Ridge (L2), Lasso (L1), and Elastic Net (a combination of both)—to prevent overfitting and handle multicollinearity. These models are beneficial in time-invariant survival analysis for selecting relevant covariates while improving model generalization, especially with high-dimensional data.
- Recursive partitioning (LTRCtrees/Rpart): Uses decision trees to model survival data, splitting the dataset based on covariates to form homogeneous subgroups. It is advantageous due to its interpretability and ability to handle nonlinear relationships without making parametric assumptions.

We initially trained each model using default hyperparameters as a baseline, followed by hyperparameter tuning through a 10-fold cross-validation over a grid of parameter combinations. The optimal set of hyperparameters was selected based on the highest Harrell’s concordance index (C-index) score, reflecting the study’s primary objective of ranking patients by treatment interruption risk. The optimal parameter sets were then used for final model training. All analysis and modeling were performed using R Statistical Software (version 4.4.0; R Foundation for Statistical Computing).

### Model Evaluation

We evaluated model performance using an 80:20 train-test split (including the Cox models to ensure comparability). Likewise, a 10-fold cross-validation was performed on the train set, and models were trained on k-1 folds and validated on the remaining fold, repeating this process 10 times, with each fold serving as the validation set once.

Final model evaluation and comparison were performed using the C-index, a widely used metric for assessing the performance of survival models. The C-index measures the model’s ability to correctly rank pairs of observations based on survival times, with values ranging from 0.5 (random prediction) to 1.0 (perfect ranking). We calculated the C-index for each model on both the training and test sets to evaluate predictive accuracy. This approach ensured that the models performed well during training and on validation data, providing a reliable measure of generalizability. Furthermore, to assess the sensitivity and uncertainty of the predictive performance measures, we conducted 200 bootstrap resampling iterations to estimate the IQR distribution of the scores for our best performing model.

### Model Interpretability

To interpret our model predictions, we used the DALEX package in R [15] and applied 2 levels of model-agnostic explanation, that is, model level global explanations—using permutation variable importance and partial dependence plots, and individual-level explanations—using variable attribution—breakdown and SHAP values and ceteris paribus (CP) analysis.

### Global-Level Explanations

The global-level explanations are listed as follows:

1. Variable importance: Using a permutation-based approach, we assessed how removing the effect of selected variables through multiple permutations impacts model performance.
2. Partial dependence profiles: We explored the effects of selected variables by examining how the model’s predicted values change as a function of individual variables, based on randomly selected observations.

### Individual-Level Explanations

The individual-level explanations are listed as follows:

1. Variable attributions: This explains how a model’s prediction for a single observation differs from the average prediction and distributes the difference among explanatory variables. We applied two methods: (1) Breakdown additive attribution, a procedure that decomposes the model’s prediction into contributions from each explanatory variable. (2) SHAP, the averaging of variable attribution values over all (or several) possible orderings to show their individual impacts.
2. CP profiles: This explores how the model’s prediction changes when the value of a single explanatory variable is altered, while keeping all other variables constant.

### Ethical Considerations

This study was reviewed and approved as nonhuman subject research by the institutional review boards of the University of Maryland, Baltimore (HP-00108126), and the Aga Khan University Nairobi (2023/ISERC-94 (v2)). The analysis used secondary EMR data that were fully deidentified prior to receipt by the study team in accordance with Kenya’s Data Protection Act and institutional guidelines; therefore, written informed consent was not required.

## Results

**Figure 1.**
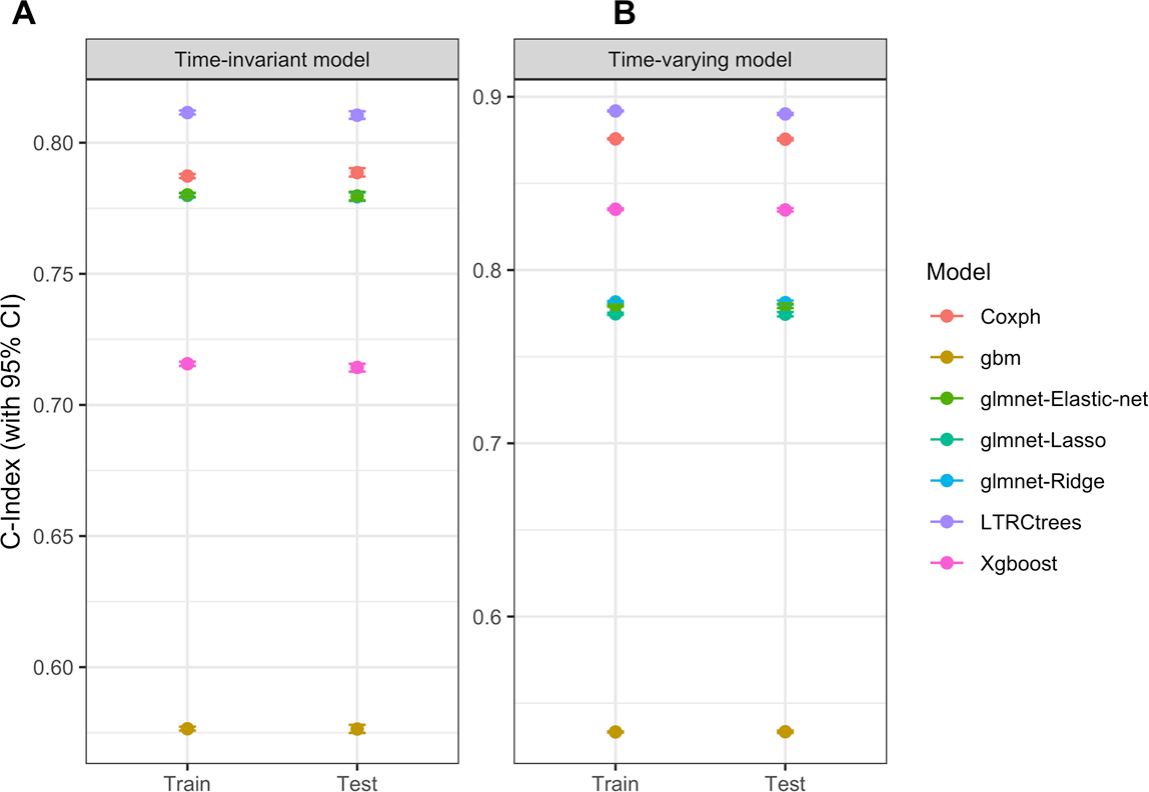
Models’ comparison: C-Index with 95% CIs. (A) C-Index comparison for the time-invariant models. (B) C-Index comparison for the time-varying models.

Our dataset comprised 621,115 unique patients on ART, with 432,041 first treatment interruption events and 959,170 multiple treatment interruption events. Data were split into an 80:20 ratio for the training and test sets, respectively. The hyperparameters for each model, along with the final parameter combinations used in the final model, are shown in Table 1. The predictive performance of ML models and their comparison with the CPH are shown in Table 2 and Figure 1, summarized by the estimated C-index scores and 95% CIs for both the train and test sets.

**Table 1.** Hyperparameters for the machine learning models.

Model (R package) and parameter	Chosen value (time-invariant model)	Chosen value (time-varying model)
Cox proportional hazards (survival)		
N/A^a^	N/A	N/A
Gradient boosting machine (gbm3)		
distribution	coxPH (ties = Efron)	coxph (ties = Efron)
n.trees	100	3000
interaction.depth	3	3
n.minobsinnode	5	20
shrinkage	0.01	0.001
bag.fraction	1	0.5
cv.folds	10	10
id	Unique value of patient ID	Unique value of patient ID
Extreme gradient boosting (xgboost)		
Objective	survival:cox	survival:aft—accelerated failure time
eval_metric	cox-nloglik	aft-nloglik
max_depth	5	6
eta	0.2	0.3
colsample_bytree	0.8	1
min_child_weight	5	1
subsample	1	1
n_folds	10	10
best nrounds	99	N/A
aft_loss_distribution	N/A	normal
aft_loss_distribution_scale	N/A	1
Regularized generalized linear models (glmnet)		
lambda	Length of 100, ranging logarithmically from 10^10^ to 10^−2^. With minimum lambda = c (Ridge: 0.01, Lasso: 0.023, Elastic Net: 0.01748)	Length of 100, ranging logarithmically from 10^10^ to 10^−2^. With minimum lambda=c (Ridge: 0.01, Lasso: 0.01, Elastic Net: 0.01)
type.measure	“C”—Harrel’s concordance measure	“C”—Harrel’s concordance measure
nfolds	10	10
maxit	1.00E+05	1.00E+05
alpha	c (0, 0.5, 1) where 0—Ridge penalty, L2 regularization only^b^. 0.5—Elastic-Net, L1 and L2 regularization^c^. 1—Lasso, L1 regularization only^d^.	c (0, 0.5, 1) where 0—Ridge penalty, L2 regularization only^b^. 0.5—Elastic-Net, L1 and L2 regularization^c^. 1—Lasso, L1 regularization only^d^.
Recursive partition (LTRCtrees/rpart)		
complexity parameter	0.0001	0.0214
xval (cross validations)	10	10
maxsurrogate	5	0
maxdepth	20	30
minsplit	20	10
maxcompete	4	4

a N/A: not applicable.

b Ridge—applies L2 regularization which penalizes the sum of the squared coefficients and is effective in cases where multicollinearity is present in the predictor variables.

c Elastic-Net regression—applies a combination of L1 and L2 regularization.

d Lasso—applies L1 regularization which can shrink some coefficients to zero, effectively performing feature selection.

**Table 2.** Models’ comparison of the time-invariant and time-varying models, using C-index scores for train and test sets.

	Time-invariant model		Time-varying model	
Model	C-Index—train (95% CI)	C-Index—test (95% CI)	C-Index—train (95% CI)	C-Index—test (95% CI)
Coxph	0.7873 (0.7865‐0.7881)	0.7886 (0.7871‐0.7903)	0.8757 (0.8754‐0.8761)	0.8755 (0.8748‐0.8762)
glmnet—Lasso	0.7801 (0.7793‐0.7809)	0.7797 (0.7780‐0.7813)	0.7747 (0.7741‐0.7754)	0.7745 (0.7732‐0.7758)
glmnet—Ridge	0.7799 (0.7791‐0.7807)	0.7794 (0.7778‐0.7810)	0.7816 (0.7810‐0.7821)	0.7812 (0.7800‐0.7824)
glmnet—Elastic-net	0.7801 (0.7793‐0.7809)	0.7796 (0.7780‐0.7812)	0.7795 (0.7789‐0.7801)	0.7793 (0.7780‐0.7805)
gbm	0.5765 (0.5758‐0.5773)	0.5764 (0.5749‐0.5780)	0.5334 (0.5330‐0.5337)	0.5335 (0.5328‐0.5342)
Xgboost	0.7157 (0.7149‐0.7165)	0.7143 (0.7127‐0.7157)	0.8351 (0.8346‐0.8356)	0.8347 (0.8338‐0.8357)
LTRCtrees	0.8115 (0.8108‐0.8123)	0.8105 (0.8091‐0.8120)	0.8918 (0.8915‐0.8921)	0.8901 (0.8895‐0.8907)

In the time-invariant analysis, the glmnet models—Lasso, Ridge, and Elastic-Net—produced comparable results to the CPH model, with train C-index values ranging from 0.7799 to 0.7801 and test C-index values ranging from 0.7794 to 0.7796. XGBoost performed slightly worse than the CPH model, while the GBM model had the lowest performance. The LTRCtrees model outperformed all other ML models and the CPH model with a C-index value of 0.8115 for the train set and 0.8105 for the test set. The LTRCtrees model remained the top performer in the time-varying analysis with a C-index of 0.8918 for the train set and 0.8901 for the test set. XGBoost was the next best performing ML model and slightly comparable with the CPH model. We further evaluated the best-performing model (ie, LTRCtrees), using a 200-bootstrap resamples; the C-index interquartile range remained stable across resamples.

**Figure 2.**
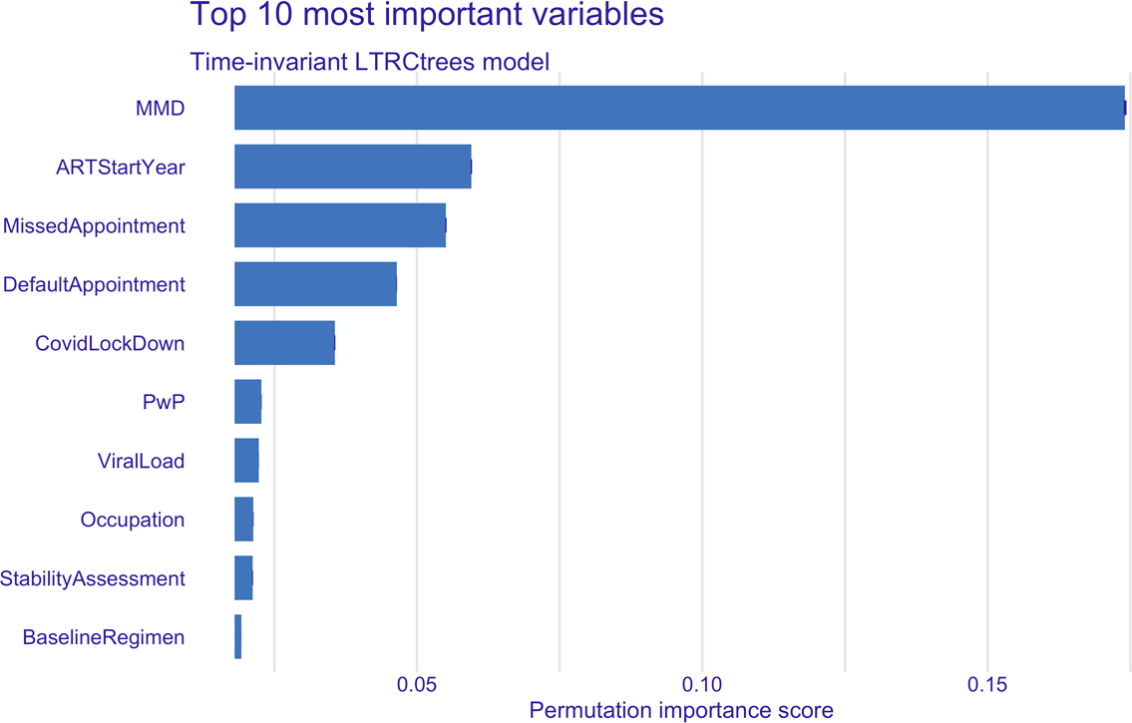
LTRCtree time-invariant model top 10 most important variables. ART: antiretroviral therapy; MMD: multimonth dispensing; PwP: prevention with positives package.

Figures2  3 show the permutation-based variable importance for the top 10 variables in both the time-invariant and time-varying models. MMD emerged as the most important variable in the time-invariant model, while the duration of treatment interruption was most important in the time-varying model. In addition, variables such as viral load, ART start year, stability assessment, baseline regimen, and occupation were consistently ranked among the top 10 most influential variables in both models. The permutation-based variable importance results were similar to the ranking of the absolute values of the coefficients of the CPH models (Table 3). MMD, missed and defaulted appointments, year of ART initiation, occupation, and stability assessment consistently ranked among the top 10 variables in both the time-invariant LTRCtree and CPH models. In the time-varying models, duration of interruption, MMD, year of ART initiation, baseline regimen, and occupation were consistently top influencers in both LTRCtree and CPH models.

**Figure 3.**
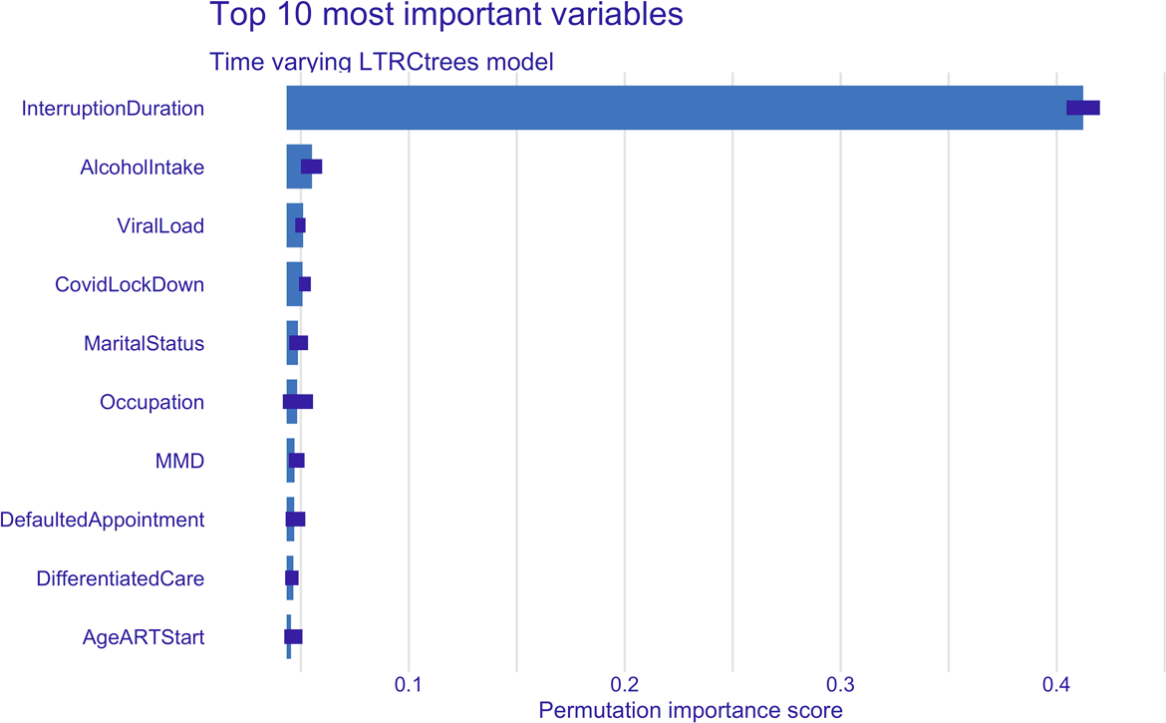
LTRCtree time-varying model top 10 most important variables. ART: antiretroviral therapy; MMD: multimonth dispensing.

**Table 3.** Hazard ratio of the top 10 variables from the time-invariant and time-varying Cox proportional hazards models.

Covariates	Time invariant—first treatment interruption		Time varying—multiple treatment interruption	
	N=497,208; events=346,118^a^		N=7,352,030; events=842,500	
	Hazard ratio (95% CI)^b^	*P* value	Hazard ratio (95% CI)^c^	*P* value
Marital status				
Other	Reference category		Reference category	
Cohabiting	1.02 (0.97-1.07)	.39	1.02 (0.99-1.05)	.26
Divorced	1.00 (0.95-1.04)	.91	0.98 (0.95-1.01)	.21
Married, monogamous	1.00 (0.95-1.05)	.89	0.99 (0.96-1.02)	.45
Married, polygamous	1.07 (1.02-1.12)	.01	0.86 (0.84-0.89)	<.001
Separated	1.10 (1.03-1.18)	<.001	1.18 (1.13-1.23)	<.001
Single	1.06 (1.01-1.11)	.02	1.01 (0.98-1.04)	.45
Widowed	1.07 (1.02-1.13)	<.001	1.00 (0.97-1.03)	.91
Employment status				
Employed	Reference category		Reference category	
Unemployed	1.00 (0.99-1.01)	.72	1.00 (0.99-1.01)	.75
Unknown	1.34 (1.33-1.36)	<.001	1.24 (1.23-1.25)	<.001
Adherence				
Good	Reference category		Reference category	
Fair	1.26 (1.23-1.29)	<.001	0.97 (0.95-0.99)	<.001
Bad	1.81 (1.69-1.94)	<.001	1.09 (1.03-1.15)	<.001
Unknown	1.13 (1.10-1.17)	<.001	0.95 (0.93-0.97)	<.001
Alcohol intake				
Never	Reference category		Reference category	
Monthly or less	0.93 (0.89-0.97)	<.001	0.98 (0.95-1.00)	.08
2-4 times a month	0.95 (0.89-1.02)	.13	1.02 (0.98-1.07)	.26
2-3 times a week	0.99 (0.94-1.05)	.85	1.02 (0.98-1.05)	.36
4 or more times a week	0.96 (0.91-1.01)	.09	1.01 (0.98-1.04)	.59
Unknown	0.99 (0.95-1.03)	.53	1.18 (1.15-1.21)	<.001
Viral load				
<200	Reference category		Reference category	
200‐999	1.10 (1.09-1.11)	<.001	1.00 (1.00-1.01)	.27
>1000	1.14 (1.12-1.16)	<.001	1.10 (1.09-1.11)	<.001
Unknown	0.82 (0.81-0.83)	<.001	1.11 (1.10-1.11)	<.001
Clinical stability assessment				
Stable	Reference category		Reference category	
Unstable	1.31 (1.30-1.32)	<.001	1.09 (1.08-1.09)	<.001
Baseline ART^d^ regimen				
Other	Reference category		Reference category	
2NRTIs^e^ + Boosted PI^f^.	0.85 (0.83-0.87)	<.001	0.89 (0.88-0.91)	<.001
2NRTIs + INSTI^g^.	1.11 (1.08-1.13)	<.001	1.02 (1.01-1.04)	<.001
2NRTIs + NNRTI^h^	1.59 (0.79-3.17)	.19	0.99 (0.71-1.39)	.97
NRTI + INSTI	1.14 (1.04-1.25)	.01	1.30 (1.24-1.36)	<.001
Multimonth dispensing (months)				
1	Reference category		Reference category	
2	0.57 (0.57-0.58)	<.001	0.75 (0.75-0.76)	<.001
3‐5	0.28 (0.28-0.28)	<.001	0.46 (0.46-0.46)	<.001
6	0.15 (0.14-0.15)	<.001	0.27 (0.27-0.28)	<.001
6+	0.04 (0.04-0.05)	<.001	0.22 (0.21-0.22)	<.001
Prevention with positives package				
0	Reference category		Reference category	
1	0.86 (0.85-0.86)	<.001	0.93 (0.92-0.94)	<.001
2	0.61 (0.60-0.62)	<.001	0.85 (0.84-0.86)	<.001
3 or more	0.59 (0.59-0.60)	<.001	0.85 (0.84-0.85)	<.001
ART initiation year				
2017	Reference category		Reference category	
2018	1.21 (1.20-1.22)	<.001	1.20 (1.19-1.21)	<.001
2019	1.50 (1.48-1.52)	<.001	1.48 (1.47-1.49)	<.001
2020	1.68 (1.66-1.71)	<.001	1.83 (1.82-1.85)	<.001
2021	2.02 (1.99-2.05)	<.001	2.22 (2.20-2.25)	<.001
2022	2.08 (2.05-2.12)	<.001	2.64 (2.61-2.68)	<.001
2023	0.63 (0.61-0.65)	<.001	3.51 (3.42, 3.60)	<.001
COVID-19 pandemic period				
Non–COVID-19 period	Reference category		Reference category	
COVID-19 period	1.17 (1.16-1.18)	<.001	0.92 (0.91-0.92)	<.001
Prior missed appointment				
No	Reference category		N/A^i^	N/A
Yes	0.35 (0.34-0.35)	<.001	N/A	N/A
Prior default appointment				
No	Reference category		N/A	N/A
Yes	0.52 (0.51-0.53)	<.001	N/A	N/A
Missed appointments				
0	N/A	N/A	Reference category	
1-3	N/A	N/A	0.93 (0.93-0.94)	<.001
4-6	N/A	N/A	0.81 (0.80-0.82)	<.001
7+	N/A	N/A	0.73 (0.72-0.75)	<.001
Default appointments				
0	N/A	N/A	Reference category	
1-3	N/A	N/A	1.00 (1.00-1.01)	.1
4-6	N/A	N/A	0.97 (0.96-0.98)	<.001
7+	N/A	N/A	0.95 (0.94-0.96)	<.001
Duration of treatment interruptions				
0 (No interruptions)	N/A	N/A	Reference category	
<30 days	N/A	N/A	29.94 (29.63-30.26)	<.001
30-197 days	N/A	N/A	19.63 (19.51-19.75)	<.001
180-364 days	N/A	N/A	8.15 (8.08-8.23)	<.001
365 days or more	N/A	N/A	2.85 (2.82-2.88)	<.001

a N=number of observations in the train dataset, that is, 80% of the entire dataset; events=number of treatment interruptions.

b Concordance=0.79 (se=0).

c Concordance=0.88 (se=0).

d ART: antiretroviral therapy.

e NRTIs: nucleoside analog reverse transcriptase inhibitors.

f PI: protease inhibitor.

g INSTI: integrase strand transfer inhibitor.

h NNRTI: nonnucleoside analog reverse transcriptase inhibitor.

i N/A: not applicable.

**Figure 4.**
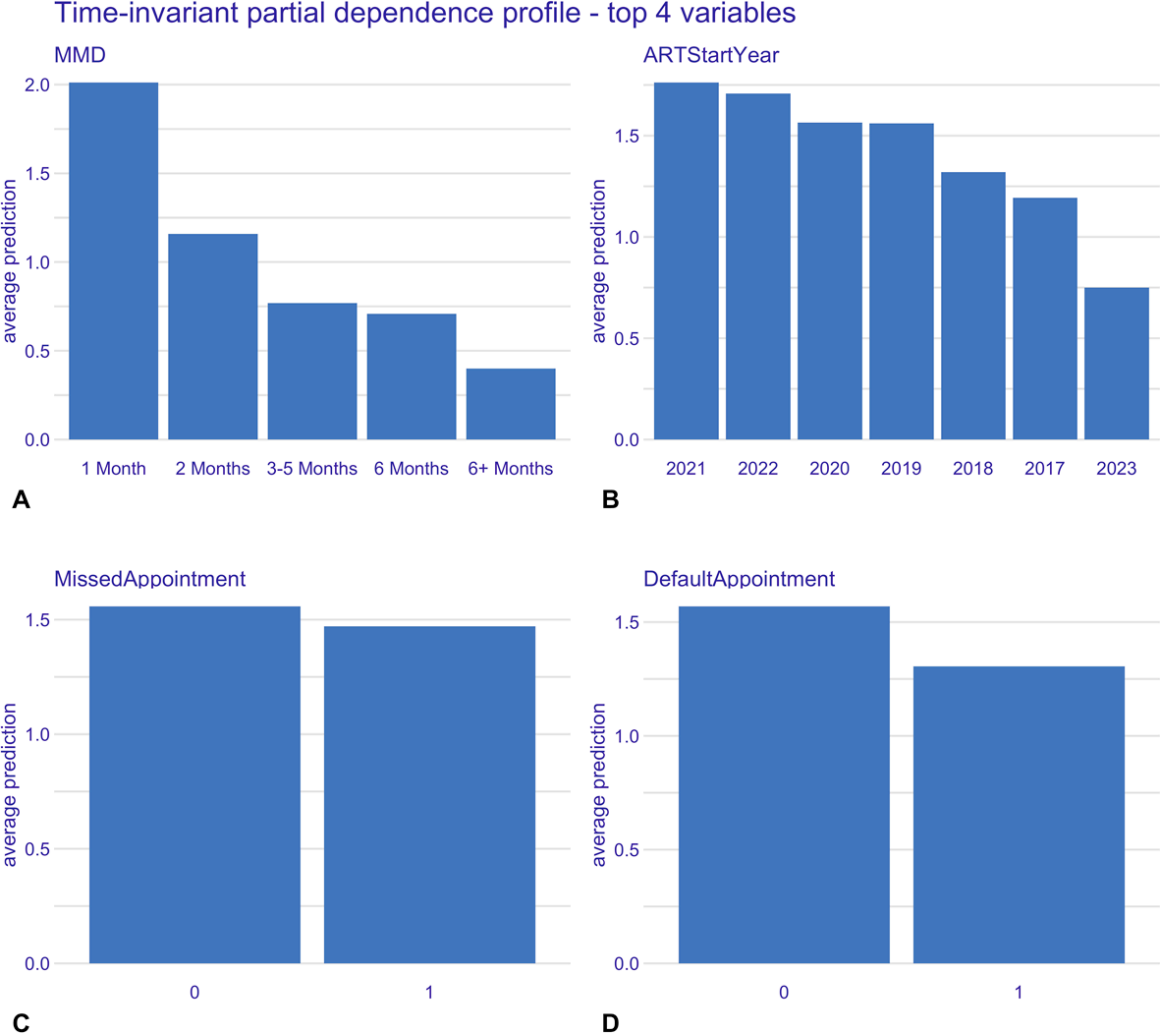
Time-invariant model; partial dependence plots for top 4 variables. Panels show the marginal effect of each variable on the average predicted risk of treatment interruption. (A) Multimonth dispensing duration, (B) ART initiation year, (C) missed appointment, and (D) defaulted appointment. ART: antiretroviral therapy; MMD: multimonth dispensing.

The full results of the time-invariant and time-varying CPH models are included in Table S2 in Multimedia Appendix 1. The partial dependence plots for the top 4 variables from both models are displayed in Figures4  5. These plots show the average predicted risk for each of these variables. Short dispensing interval (MMD of 1 month), initiating ART in 2021, and having not missed or defaulted on an appointment were associated with higher predicted risks of first treatment interruption. Conversely, longer dispensing intervals (6+ months MMD), initiating ART in 2023, and having missed or defaulted on an appointment had a lower predicted risk of first treatment interruption. Short interruption durations (<30 days), unknown viral load, separated marital status, and unspecified alcohol use were all associated with higher predicted risk of multiple treatment interruption, while having no prior interruptions (ie, interruption duration of zero), lower viral load (<200), and no alcohol consumption showed lower predicted risk of multiple treatment interruption.

**Figure 5.**
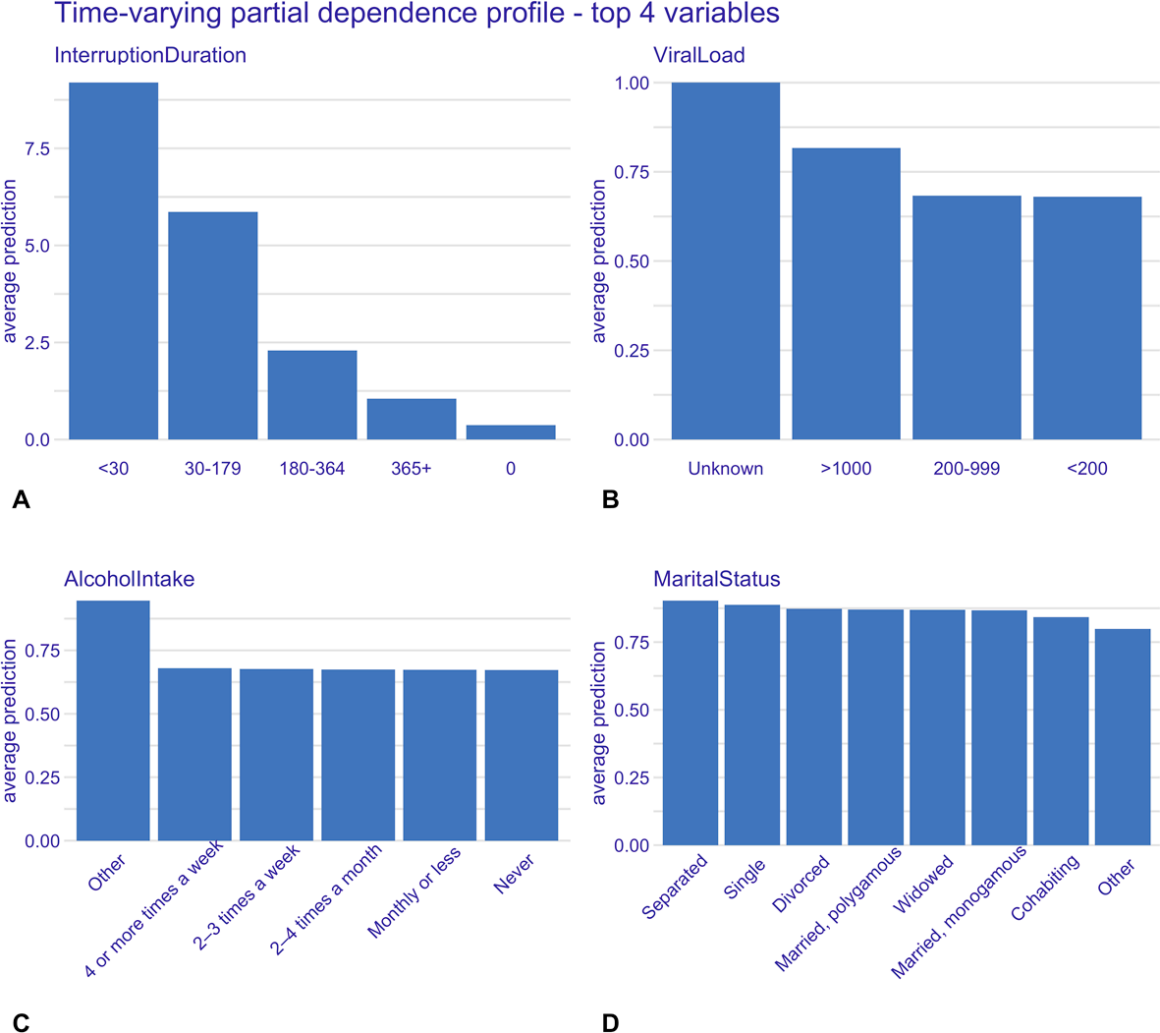
Time-varying model; partial dependence plots for top 4 variables. Panels show the marginal effect of each variable on the average predicted risk of treatment interruption. (A) Treatment interruption duration, (B) viral load count, (C) alcohol use, and (D) marital status.

To examine individual-level predictions for the time-invariant model, we randomly selected 2 observations from the dataset: one representing a patient (labeled “patient A”) who experienced a first treatment interruption, and another representing a patient (labeled “patient B”) who did not experience an interruption by the end of the follow-up period.

**Figure 6.**
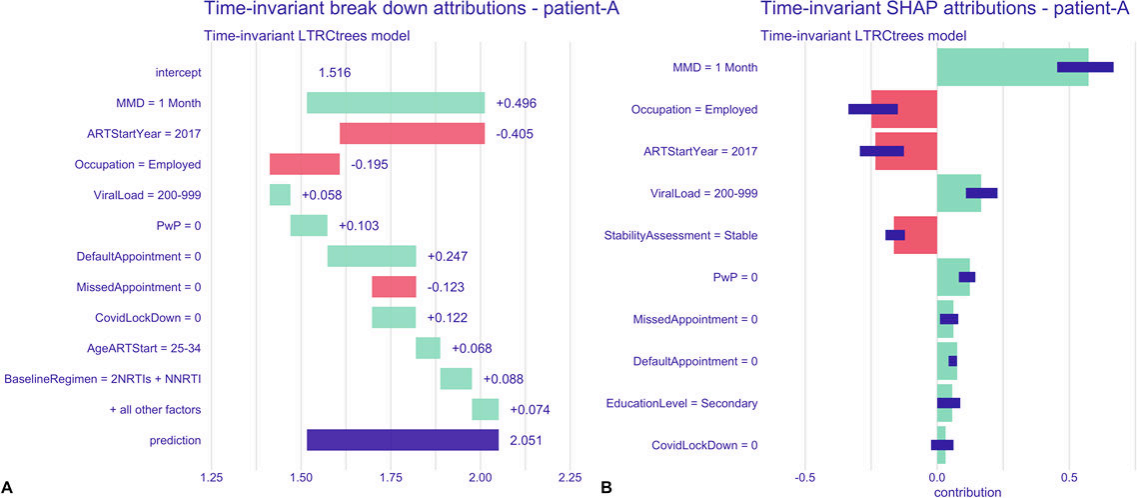
Time-invariant model breakdown and SHAP attribution plots for patient A. **(A)** Breakdown plot decomposing the model prediction into cumulative covariate contributions. Intercept—model’s overall average prediction value; green bars, with (+) sign indicate increased risk of treatment interruption; red bars, with (−) sign indicate decreased risk of interruption; and blue bar, “prediction” is the individual predicted risk of interruption for the patient. **(B)** SHAP plot summarizing the direction and magnitude of individual covariate effects on the predicted risk of treatment interruption. Note: variable labels shown in the plots represent categorical levels of the corresponding covariates; see Table S1 in Multimedia Appendix 1 for full description. ART: antiretroviral therapy; MMD: multimonth dispensing; NNRTI: nonnucleoside analog reverse transcriptase inhibitor; NRTIs: nucleoside analog reverse transcriptase inhibitors; PwP: prevention with positives package; SHAP: Shapley Additive Explanations.

**Figure 7.**
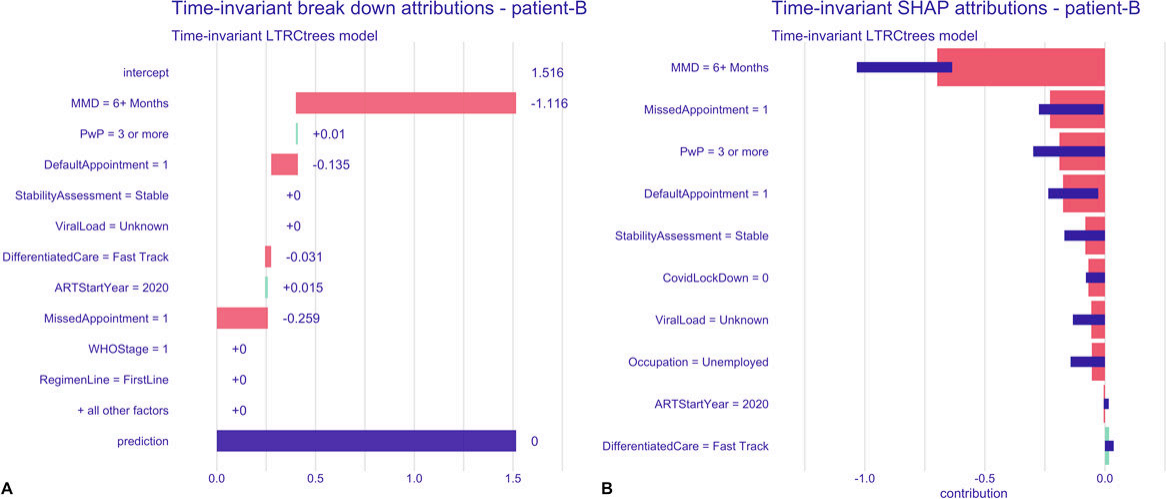
Time-invariant model breakdown and SHAP attribution plots for patient B. **(A)** Breakdown plot decomposing the model prediction into cumulative covariate contributions. Intercept—model’s overall average prediction value; green bars, with (+) sign indicate increased risk of treatment interruption; red bars, with (−) sign indicate decreased risk of interruption; and blue bar, “prediction” is the individual predicted risk of interruption for the patient. **(B)** SHAP plot summarizing the direction and magnitude of individual covariate effects on the predicted risk of treatment interruption. ART: antiretroviral therapy; MMD: multimonth dispensing; PwP: prevention with positives package; SHAP: Shapley Additive Explanations.

The row labeled “intercept” represents the overall average prediction value (1.516) for the model—baseline prediction (Figure 6A). The subsequent rows show how the mean prediction changes when a specific covariate is fixed. Green bars indicate an increase in the risk of treatment interruption, while red bars show a decrease in the risk. The final row (prediction), shown with a blue bar, displays the overall mean value and change, giving the final predicted risk of treatment interruption for the patient. Patient A had a high predicted risk of treatment interruption, with a risk score of 2.051, which is 0.535 points higher than the model’s average prediction (Figure 6). Covariates influencing this prediction are short MMD intervals (1 month), viral load of 200‐999, did not receive a prevention with positive package, not defaulted on appointment, non–COVID-19 pandemic period, aged 25‐34 years at the start of ART, and baseline regimen of 2NRTIs + NNRTI. Patient B, on the other hand, had a low predicted risk (0) of interruption, largely driven by the long MMD interval (6+ months), enrolled in the DSD fast-track model, and had prior missed and defaulted appointments contributing to a reduced risk (Figure 7).

The right plots of both Figures6  7 show the SHAP plots for the patients, where the SHAP values represent the computed mean attribution across 25 random orderings of the explanatory variables. The red bars indicate an average increase in risk, while the green bars show a decrease in risk. The violet box plots summarize the distribution of the attributions over the 25 orderings. The plots reveal similar influential variables as in the breakdown attributions, with a slight reordering of importance and the inclusion of additional variables—such as education level and stability assessment for patient A, and COVID-19 pandemic period for patient B.

**Figure 8.**
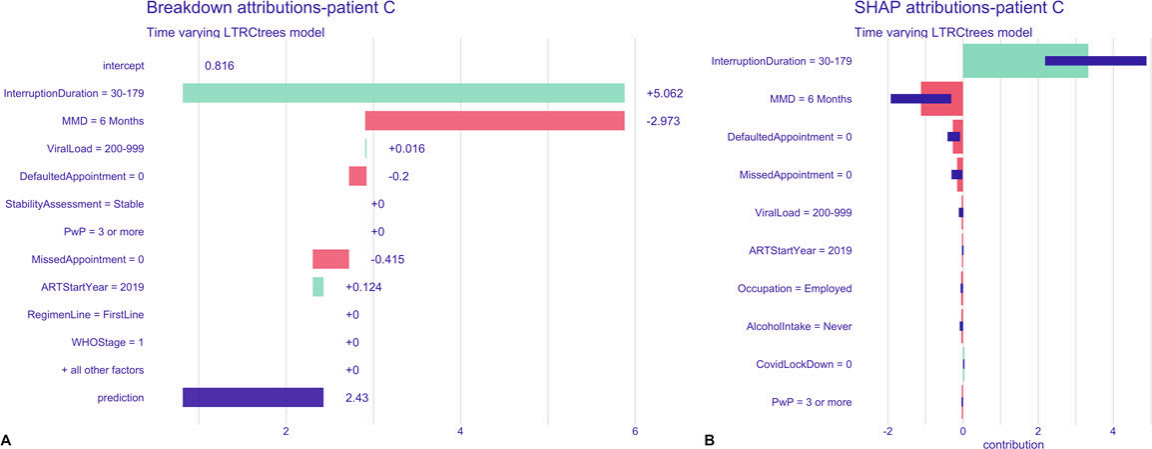
Time-varying model, breakdown, and SHAP attribution plots for patient C. **(A)** Breakdown plot decomposing the model prediction into cumulative covariate contributions. Intercept—model’s overall average prediction value; green bars, with (+) sign indicate increased risk of treatment interruption; red bars, with (−) sign indicate decreased risk of interruption; and blue bar, “prediction” is the individual predicted risk of interruption for the patient. **(B)** SHAP plot summarizing the direction and magnitude of individual covariate effects on the predicted risk of treatment interruption. ART: antiretroviral therapy; MMD: multimonth dispensing; PwP: prevention with positives package; SHAP: Shapley Additive Explanations.

**Figure 9.**
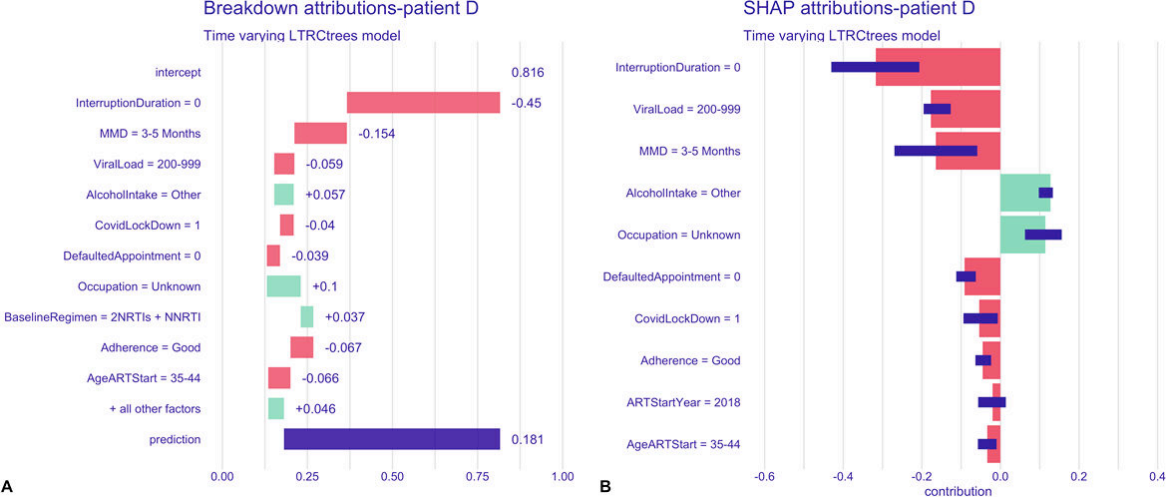
Time-varying model, breakdown, and SHAP attribution plots for patient D. **(A)** Breakdown plot decomposing the model prediction into cumulative covariate contributions. Intercept—model’s overall average prediction value; green bars, with (+) sign indicate increased risk of treatment interruption; red bars, with (−) sign indicate decreased risk of interruption; and blue bar, “prediction” is the individual predicted risk of interruption for the patient. **(B)** SHAP plot summarizing the direction and magnitude of individual covariate effects on the predicted risk of treatment interruption. ART: antiretroviral therapy; MMD: multimonth dispensing; NNRTI: nonnucleoside analog reverse transcriptase inhibitor; NRTIs: nucleoside analog reverse transcriptase inhibitors; SHAP: Shapley Additive Explanations.

To examine the time-varying model’s prediction for a single time-interval instance for a patient, we randomly selected 2 patients: patient C, who had experienced 1 or more interruptions and subsequently returned to care; patient D, who had no history of treatment interruption. Figure 6 shows the breakdown and SHAP attribution plots for a single time interval for patient C. After considering all variable contributions, the final predicted risk of subsequent treatment interruption for this patient was 2.43, which is 1.614 points above the average prediction. The key covariate contributing to this risk prediction is the patient’s previous interruption duration (duration of 30‐179 days). In contrast, being on a 6-month MMD interval and having not missed or defaulted on any appointments were associated with a lower risk of subsequent interruptions. The SHAP plot reflects similar variable contributions (Figure 8B). The predicted risk of treatment interruption for patient D was 0.181, which is 0.635 lower than the average model prediction (Figure 9A). Key covariates contributing to this reduced risk include no prior treatment interruptions (interruption duration of zero), being on a 3‐ to 5-month MMD interval, having a viral load between 200 and 999 cp/mL, the COVID-19 pandemic period, no defaulted appointments, good adherence, and starting ART between the ages of 35 and 44 years. SHAP plots (Figure 9B) further highlighted covariates associated with a lower risk for this patient.

**Figure 10.**
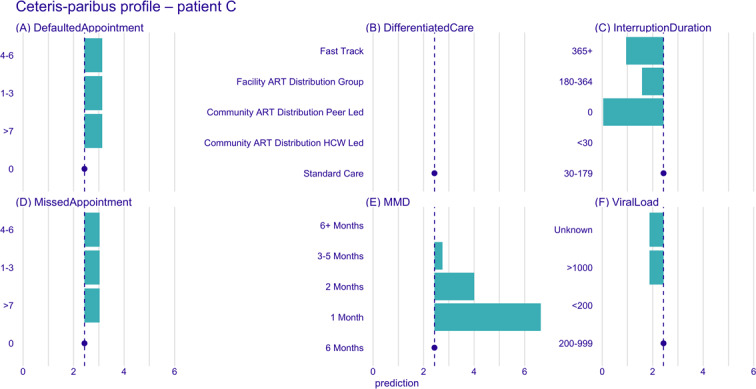
Ceteris paribus profile plots for patient C. Panels show the marginal effect of changes of each variable on the predicted risk of treatment interruption. (A) Defaulted appointments, (B) differentiated care model, (C) interruption duration, (D) missed appointments, (E) multimonth dispensing duration, and (F) viral load. Blue dot is the value of the covariate; blue dotted line corresponding prediction for the patient; green bars—predicted change in value and direction for a specific covariate; and bars right of the dotted line indicate increased risk, while those to the left indicate decreased risk. ART: antiretroviral therapy; HCW: health care worker; MMD: multimonth dispensing.

Using CP profile plots, we further examined how the model’s predictions for these patients would shift if key covariates—such as the duration of the previous interruption, viral load, DSD model, MMD, missed appointments, and defaulted appointments—were altered. This allowed us to explore how changes in time-varying covariates impact the risk of multiple treatment interruptions. The blue dot and dotted line in the CP plot (Figures10  11) represent the values of the covariates and the corresponding prediction for the patient. Each green bar in the plot shows the predicted change in value and direction for a specific covariate, with bars pointing to the right of the dotted line indicating an increased risk of interruption and bars pointing to the left indicating a decreased risk.

**Figure 11.**
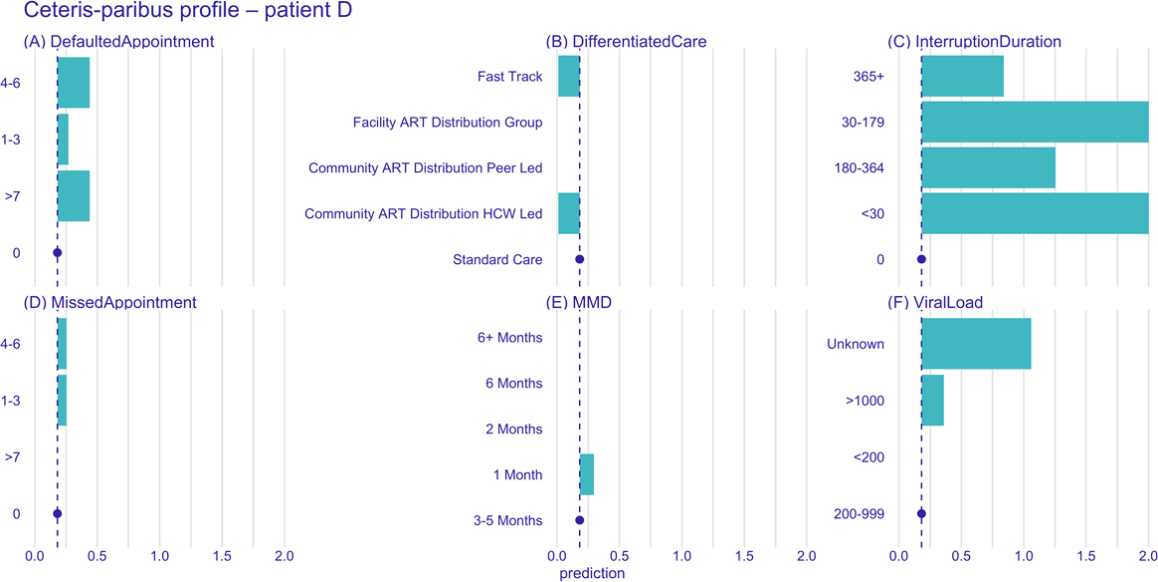
Ceteris paribus profile plots for patient D. Panels show the marginal effect of changes of each variable on the predicted risk of treatment interruption. (A) Defaulted appointments, (B) differentiated care model, (C) interruption duration, (D) missed appointments, (E) multimonth dispensing duration, and (F) viral load. Blue dot is the value of the covariate; blue dotted line corresponding prediction for the patient; green bars—predicted change in value and direction for a specific covariate; and bars right of the dotted line indicate increased risk, while those to the left indicate decreased risk. ART: antiretroviral therapy; HCW: health care worker; MMD: multimonth dispensing.

The CP plot (Figure 10) for patient C indicates that their risk of future treatment interruptions would increase significantly if they were to miss or default on an appointment or be placed on a shorter MMD interval. However, the predicted risk remains unchanged if they were to transition across different DSD models. The future risk of treatment interruption would increase for patient D if they miss or default on an appointment, had an increase in their viral load, were placed on a 1-month MMD interval, or experienced a treatment interruption, irrespective of how long it took to return to care (Figure 11). Transitioning from their current standard care DSD model to fast track or community ART distribution health care worker–led models would slightly reduce the risk of interruption.

## Discussion

### Principal Findings

We compared the traditional CPH model with 6 different ML models to predict the risk of treatment interruption in 621,115 patients receiving ART in 256 facilities across all 47 counties in Kenya. The analysis was conducted in 2 parts: time-invariant models for predicting time-to-first treatment interruptions and time-varying models for predicting time-to-multiple treatment interruptions. Model evaluation was performed using an 80:20 train-test split, and Harrell’s C-index was applied to compare the performance of the models on both the train and test sets. Prediction from the best-performing model was then interpreted at both global and individual levels.

The RP model outperformed all other models in both the time-invariant and time-varying analyses. It achieved C-index values of 0.8115 for the train set and 0.8105 for the test set in the time-invariant model, and 0.8918 for the train set and 0.8901 for the test set in the time-varying model. These results align with findings from previous studies that have compared CPH models with ML models, demonstrating the effectiveness of RP models in survival analysis. Previous studies have reported C-index values ranging from 0.63 to 0.96, primarily from ML models such as GBM, random survival forest, and survival support vector machines in predicting survival for patients with cancer [16-19]. Other studies that focused on predicting treatment interruptions in patients on ART had typically reported metrics such as sensitivity, specificity, and area under the curve, with values ranging between 61.9% and 76.0% [20-24]. Few of these studies have incorporated time-varying covariates [16,25]. Even when compared with prior studies that evaluated ML models against CPH [17], our RP approach achieved higher discrimination, with C-index values of 0.8105 and 0.8901 compared with reported XGBoost performance of approximately 0.73. Notably, our study stands out as one of the few to apply RP within a time-varying survival framework, effectively constructing a survival tree that accommodates time-varying covariates.

The RP method offers flexibility in detecting complex interactions without assuming proportional hazards. They automatically select covariates and split points, allowing more adaptive modeling of survival times [26]. This method is advantageous for identifying nonlinear relationships and interactions, making it particularly useful in our case where covariate effects changed over time. The RP model also had the advantage of simple hyperparameter tuning and faster computational times compared with the other ML models we evaluated. We attempted to train a random survival forest model but were unsuccessful due to the large computational resources and extensive time required to handle our large dataset. Likewise, the widely used randomForestSRC R package does not currently support time-varying covariates directly. While random forests are known for their high predictive performance, their significant computational requirements make them less practical for real-world applications [16].

To ensure that our results were fully comparable with the traditional CPH, we implemented a cohesive framework for explaining the ML predictions with meaningful insights. Using a model-agnostic [15] explainer framework, we gained detailed insights into how specific variables influenced individual-level predictions and overall global outcomes, similar to the interpretations provided by hazard ratios in the CPH model. The explainer framework offers additional advantages by capturing complex, nonlinear relationships that traditional CPH may overlook.

On a global level, the permutation-based variable importance from the RP model was similar to the ranking of the absolute values of the coefficients of the CPH model for both time-invariant and time-varying models. In addition, the effects of the selected covariates we considered in the partial dependence plots were consistent with the direction of hazard ratios in the CPH model. This combination of techniques offers diverse insights into our model predictive performance. They reveal the most significant effects and risks associated with treatment interruption across various covariates without the assumptions of linearity, hence capturing potential nonlinear effects that traditional CPH models might overlook. Overall, these methods provide more comprehensive global explanations, particularly for complex variable interactions.

Further exploration using breakdown attribution and SHAP plots reveals how individual variables influence each patient’s predicted risk within specific time intervals. On a global level, the time-invariant model was primarily influenced by MMD. Considering this at the patient level, the breakdown and SHAP plots provide detailed insights into how MMD increases or decreases risk, explaining why patient A was at a higher risk than patient B based on MMD. In addition, these plots highlight the most influential covariates for predicting the risk of a first treatment interruption for each patient, emphasizing individual variability in risk factors. For the time-varying model, we furthered the instance-level exploration using CP profiles. These plots provided insights into how a patient’s predicted risk would change if certain variables were adjusted. For example, with patient C—who has no history of treatment interruption—we explored the predicted risk of an interruption if the patient were to miss or default on an appointment, transition to a less intensive DSD model, or receive a different drug-dispensing interval. In contrast, for patient D—a patient with a history of interruptions—the focus was on understanding how the predicted risk of another interruption might change if they transitioned to a less intensive care model, adjusted their dispensing interval, or missed an appointment. The changes highlighted for each patient provide actionable insights to guide transitions between DSD models and targeted interventions to prevent treatment interruptions. Following ART initiation, CP profiles can identify patients whose interruption risk under standard care would decrease with earlier DSD enrollment or longer dispensing intervals. For patients returning to care after an interruption, CP profiles quantify how changes in follow-up intensity or MMD duration influence recurrent interruption risk, supporting tailored rather than routine care. These scenario-based insights align with recent World Health Organization and global guidance [27,28] emphasizing person-centered, targeted care packages for patients reengaging in treatment.

Bringing the various components together, our model could be integrated into an EMR system to predict a patient’s risk of treatment interruption in real time. This integration would allow the model to continuously process each patient’s historical data, current visit date, and next scheduled visit date, creating a new data point for each interaction. With each visit, the EMR system would automatically update risk predictions, enabling health care providers to proactively identify high-risk patients before interruptions occur. The Kenya EMR currently includes an ML module that generates interruption risk scores based on a limited set of patient-centric covariates. By leveraging our model’s broader range of covariates, the current system can be optimized for improved prediction accuracy and comprehensive risk assessment.

### Limitations

Our study had some limitations. First is the issue of missing data, as highlighted in our previous work [6]. This was addressed by creating an “unknown” category for affected variables, given that the data were not missing at random. Variables with more than 60% missingness, such as baseline and follow-up CD4 counts, were excluded, which may have introduced bias. Sensitivity analyses comparing complete-case models with alternative “unknown” category specifications were conducted in prior work [6] and showed consistent results. Multiple imputation was not considered, given data were not missing at random. Second, while the explainer we used is model-agnostic, it lacks built-in support for survival-specific metrics such as Brier score and area under the curve. Survex explainer is specifically designed for survival models, but it currently does not support the LTRCtrees package or time-varying survival models. A potential work-around could be treating the model as a classification task, focusing on whether there was an interruption or not within a specified fixed time, although this approach will sacrifice the temporal dimension of survival analysis or future studies can consider implementing other Survex-supported algorithms with the ability to fit time-varying covariates. Finally, generating breakdown and SHAP attribution profiles for individual predictions was computationally intensive, particularly for SHAP, which may limit real-time EMR use. However, subsampling across observations or feature permutations can substantially reduce computational cost while preserving accurate SHAP approximations.

### Conclusions

Our study compared the predictive performance of the traditional Cox model with 6 different ML models for predicting the risk of treatment interruptions in patients on ART. Overall, our results demonstrate that ML models outperform the traditional Cox model, with RP, in particular, effectively capturing nonlinear effects and complex interactions without relying on proportional hazards assumptions, making it well suited for time-varying survival analyses. Our model-agnostic explanation aligns well with the Cox model results while also offering additional, patient-specific insights for targeted interventions. The combination of enhanced predictive performance and interpretability demonstrates how ML models can effectively support patient-centered care strategies to reduce the likelihood of treatment interruptions in ART.

## Supplementary material

10.2196/78964 Multimedia Appendix 1 Full list of covariates and descriptions.

## Abbreviations

ART: antiretroviral therapy
C-index: concordance index
CP: ceteris paribus
CPH: Cox proportional hazards
DSD: differentiated service delivery
EMR: electronic medical record
GBM: gradient boosting machine
ML: machine learning
MMD: multimonth dispensing
RP: recursive partitioning
SHAP: Shapley Additive Explanations
XGBoost: extreme gradient boosting
