# Supplementary material for: Prediction of First and Multiple Antiretroviral Therapy Interruptions in People Living With HIV: Comparative Survival Analysis Using Cox and Explainable Machine Learning Models
Source: JMIR Med Inform. 2026 Feb 4;14:e78964. doi: 10.2196/78964 (PMC12871577; doi:10.2196/78964)
Supplement: Multimedia Appendix 1 [file medinform-v14-e78964-s001.docx]

Multimedia Appendix 1

Table S1. Full list of covariates and descriptions.

| **Characteristic** | **Description** | **Categories** |
| --- | --- | --- |
| Age group at ART start | Age category when patients started ART | 0–14; 15–24; 25–34; 35–44; 45–59; 60+ |
| Gender | Sex assigned to the patient at birth | Female; Male |
| Education Level | The education level of the patient | Primary; Secondary; Tertiary; None; Unknown |
| Marital Status | Marital Status of the patient | Cohabiting; Divorced; Married Monogamous; Married Polygamous; Separated; Single; Widowed; Other; Unknown |
| Employment Status | Patient employment status | Employed; Unemployed; Unknown |
| Non-communicable disease (NCDs) | Patient has one or more of the following non-communicable diseases, i.e. Chronic respiratory disease, Cardiovascular Disease, Cancer, and Diabetes. | 0 -No; 1- Yes |
| Adherence | Patients ART Adherence rate based on pill counts. Calculated from the % of medication taken: >= 95% - Good  85-94% - Fair  <85% - Bad | Bad; Fair; Good; Unknown |
| Alcohol intake | Patient alcohol consumption assessment | 2 To 3 Times A Week; 2 To 4 Times A Month; 4 Or More Times A Week; Monthly or Less; Never; Unknown |
| Baseline ART Regimen | Patient baseline regimen base | 2NRTIs + Boosted PI; 2NRTIs + INSTI; 2NRTIs + NNRTI; NRTI + INSTI; Other |
| Regimen Line | Patient drug regimen | First Line; Second Line; Third Line; Other |
| ART Initiation Year | Year ART was initiated | Years 2017 to 2023 |
| WHO Stage | WHO staging of patient | I; II; III; IV; Unknown |
| Viral Load | Viral Load test category (in cp/ml) | <200; 200-999; >1000; Unknown1 |
| Clinical Stability Assessment | Clinical Stability assessment of the patient. Clinically stable is defined as On ART for at least 12 months, showing no signs or symptoms of WHO Clinical Stage 2,3 or 4 and virally suppressed (i.e. <50 cp/ml or 50-199 cp/ml) | Stable; Unstable; Unknown |
| Multi-month dispensing (MMD) | Multi-month dispensing (MMD) categories | 1 Month; 2 Months; 3-5 Months; 6 Months; 6+ Months |
| Differentiated Service Delivery2 | Differentiated service delivery, models of care (Facility and Community based) | Community ART Distribution HCW Led; Community ART Distribution Peer Led; Facility ART Distribution Group; Fast Track; Standard Care; Unknown |
| Prevention with Positives Package | Number of preventions with positives packages offered to patients (package offering included the following: condom use; partner/family testing; support for disclosure screened for sexually transmitted infections; screened for cervical cancer; screened for substance abuse). | 0; 1; 2; 3 or More |
| Covid Pandemic | Covid Pandemic Period (with series of lock down from 22 Mar 2020 to 22 Oct 2021) | 0- Non Covid Period; 1- Covid Period |
| Missed Appointments3 | Failed to attend scheduled visit within 1–3 days of the expected clinical contact (arriving on day 4). Cumulative counts were the categorized. | 0; 1-to-3; 4-to-6; 7+ |
| Default Appointments3 | Failed to attend scheduled visit within 4–27 days of the expected clinical contact (arriving on day 28). Cumulative counts were the categorized. | 0; 1-to-3; 4-to-6; 7+ |
| Duration of Treatment Interruptions | Average Duration of any treatment interruption before a patient returned to care.  Calculated as the average time to return to care after first or multiple interruptions divided by the cumulative sum of interruptions | Less than 30 Days; 30-to-197 Days; 180-to-364 Days; 365 Days or More |

**1**. The "Unknown" category includes new patients (on ART for less than 6 months) who were not yet due for a viral load test **2**. Refer to Supplementary Text 1, below for definitions of the various models. **3**.These variables were categorized as a binary variable in time invariant analysis, i.e. defined as a missed or defaulted appointment prior to first treatment interruption.

Abbreviations: ART - antiretroviral therapy; NNRTI - non-nucleoside analogue reverse transcriptase inhibitor; NRTI - nucleoside analogue reverse transcriptase inhibitor; INSTI - integrase Strand Transfer Inhibitor PI - protease inhibitor; HCW – health care worker

# Supplementary Text 1: Definitions of Kenya’s differentiated service delivery (DSD) models.

The various differentiated service delivery (DSD) models in Kenya [1] are defined as follows:

- Standard Care: Facility-based services for clients not yet stable on ART, including those newly initiated, with co-morbidities, unsuppressed viral loads, or adherence challenges.
- Facility ART Distribution Group: A group-based model at the health facility where patient receives ART refills every three months and peer support, with clinical reviews every six months.
- Fast Track: A streamlined facility-based model offering quick, with minimal or no wait time for ART refills, a visit is scheduled for every three months.
- Community ART Distribution (HCW-Led): led by a healthcare worker (HCW) at a community location. ART refills and psychosocial support are provided every three months by a healthcare worker in the community, with facility-based clinical reviews every six months.
- Community ART Distribution (Peer-Led): Same as HCW-led but managed by a peer instead of a healthcare worker.

Table S2. Results of the time-invariant and time-varying Cox proportional hazard models, predicting first and multiple treatment interruptions.

| Covariates | Time Invariant - First Treatment Interruption | | Time Varying - Multiple Treatment Interruption | |
| --- | --- | --- | --- | --- |
|  | n = 497, 208; Events = 346,118 ^a.^ | | n = 7, 352, 030; Events = 842,500 | |
|  | Hazard Ratio (95% CI) ^b.^ | *P-*value | Hazard Ratio (95% CI) | *P*-value |
|  |  |  |  |  |
| Age group at ART start ^c.^ |  |  |  |  |
| 0–14 | Ref ^d.^ |  | ref |  |
| 15–24 | 1.33 (1.31, 1.36) | <.001 | 1.22 (1.20, 1.23) | <.001 |
| 25–34 | 1.48 (1.45, 1.50) | <.001 | 1.22 (1.21, 1.23) | <.001 |
| 35–44 | 1.60 (1.58, 1.63) | <.001 | 1.20 (1.19, 1.22) | <.001 |
| 45–59 | 1.69 (1.66, 1.72) | <.001 | 1.19 (1.18, 1.21) | <.001 |
| 60+ | 1.68 (1.64, 1.73) | <.001 | 1.21 (1.19, 1.23) | <.001 |
| Gender |  |  |  |  |
| Female | ref |  | ref |  |
| Male | 1.11 (1.10, 1.12) | <.001 | 1.04 (1.04, 1.05) | <.001 |
| Education Level |  |  |  |  |
| Tertiary | ref |  | ref |  |
| None | 1.00 (0.99, 1.02) | 0.57 | 0.99 (0.99, 1.00) | 0.13 |
| Primary | 1.03 (1.02, 1.05) | <.001 | 1.01 (1.01, 1.02) | <.001 |
| Secondary | 1.09 (1.07, 1.11) | <.001 | 1.01 (1.00, 1.03) | 0.02 |
| Marital Status |  |  |  |  |
| Other | ref |  | ref |  |
| Cohabiting | 1.02 (0.97, 1.07) | 0.39 | 1.02 (0.99, 1.05) | 0.26 |
| Divorced | 1.00 (0.95, 1.04) | 0.91 | 0.98 (0.95, 1.01) | 0.21 |
| Married Monogamous | 1.00 (0.95, 1.05) | 0.89 | 0.99 (0.96, 1.02) | 0.45 |
| Married Polygamous | 1.07 (1.02, 1.12) | 0.01 | 0.86 (0.84, 0.89) | <.001 |
| Separated | 1.10 (1.03, 1.18) | <.001 | 1.18 (1.13, 1.23) | <.001 |
| Single | 1.06 (1.01, 1.11) | 0.02 | 1.01 (0.98, 1.04) | 0.45 |
| Widowed | 1.07 (1.02, 1.13) | <.001 | 1.00 (0.97, 1.03) | 0.91 |
| Employment Status |  |  |  |  |
| Employed | ref |  | ref |  |
| Unemployed | 1.00 (0.99, 1.01) | 0.72 | 1.00 (0.99, 1.01) | 0.75 |
| Unknown | 1.34 (1.33, 1.36) | <.001 | 1.24 (1.23, 1.25) | <.001 |
| Non-communicable disease |  |  |  |  |
| No | ref |  | ref |  |
| Yes | 1.03 (1.00, 1.06) | 0.02 | 0.97 (0.95, 0.98) | <.001 |
| Adherence |  |  |  |  |
| Good | ref |  | ref |  |
| Fair | 1.26 (1.23, 1.29) | <.001 | 0.97 (0.95, 0.99) | <.001 |
| Bad | 1.81 (1.69, 1.94) | <.001 | 1.09 (1.03, 1.15) | <.001 |
| Unknown | 1.13 (1.10, 1.17) | <.001 | 0.95 (0.93, 0.97) | <.001 |
| Alcohol intake |  |  |  |  |
| Never | ref |  | ref |  |
| Monthly or less | 0.93 (0.89, 0.97) | <.001 | 0.98 (0.95, 1.00) | 0.08 |
| 2 To 4 Times A Month | 0.95 (0.89, 1.02) | 0.13 | 1.02 (0.98, 1.07) | 0.26 |
| 2 To 3 Times A Week | 0.99 (0.94, 1.05) | 0.85 | 1.02 (0.98, 1.05) | 0.36 |
| 4 Or More Times A Week | 0.96 (0.91, 1.01) | 0.09 | 1.01 (0.98, 1.04) | 0.59 |
| Unknown | 0.99 (0.95, 1.03) | 0.53 | 1.18 (1.15, 1.21) | <.001 |
| WHO Stage |  |  |  |  |
| I | ref |  | ref |  |
| II | 1.16 (1.15, 1.18) | <.001 | 1.03 (1.02, 1.04) | <.001 |
| III | 1.17 (1.15, 1.19) | <.001 | 1.05 (1.04, 1.07) | <.001 |
| IV | 1.05 (1.00, 1.11) | 0.06 | 1.05 (1.02, 1.09) | 0.01 |
| Viral Load |  |  |  |  |
| <200 | ref |  | ref |  |
| 200-999 | 1.10 (1.09, 1.11) | <.001 | 1.00 (1.00, 1.01) | 0.27 |
| >1000 | 1.14 (1.12, 1.16) | <.001 | 1.10 (1.09, 1.11) | <.001 |
| Unknown | 0.82 (0.81, 0.83) | <.001 | 1.11 (1.10, 1.11) | <.001 |
| Clinical Stability Assessment |  |  |  |  |
| Stable | ref |  | ref |  |
| Unstable | 1.31 (1.30, 1.32) | <.001 | 1.09 (1.08, 1.09) | <.001 |
| Baseline ART Regimen |  |  |  |  |
| Other | ref |  | ref |  |
| 2NRTIs + Boosted PI ^e.^ | 0.85 (0.83, 0.87) | <.001 | 0.89 (0.88, 0.91) | <.001 |
| 2NRTIs + INSTI ^f.^ | 1.11 (1.08, 1.13) | <.001 | 1.02 (1.01, 1.04) | <.001 |
| 2NRTIs + NNRTI ^g.^ | 1.59 (0.79, 3.17) | 0.19 | 0.99 (0.71, 1.39) | 0.97 |
| NRTI + INSTI | 1.14 (1.04, 1.25) | 0.01 | 1.30 (1.24, 1.36) | <.001 |
| Regimen Line |  |  |  | - |
| Other | ref |  | ref |  |
| First Line | 0.99 (0.90, 1.08) | 0.77 | 1.27 (1.21, 1.33) | <.001 |
| Second Line | 1.52 (1.32, 1.75) | <.001 | 1.17 (1.08, 1.27) | <.001 |
| Third Line | 0.99 (0.97, 1.01) | 0.4 | 1.03 (1.02, 1.04) | <.001 |
| Multi-month dispensing |  |  |  |  |
| 1 Month | ref |  | ref |  |
| 2 Months | 0.57 (0.57, 0.58) | <.001 | 0.75 (0.75, 0.76) | <.001 |
| 3-5 Months | 0.28 (0.28, 0.28) | <.001 | 0.46 (0.46, 0.46) | <.001 |
| 6 Months | 0.15 (0.14, 0.15) | <.001 | 0.27 (0.27, 0.28) | <.001 |
| 6+ Months | 0.04 (0.04, 0.05) | <.001 | 0.22 (0.21, 0.22) | <.001 |
| Differentiated Service Delivery |  |  |  |  |
| Standard care | ref |  | ref |  |
| Community ART Distribution Healthcare worker led | 0.95 (0.89, 1.03) | 0.21 | 1.05 (1.01, 1.09) | 0.03 |
| Community ART Distribution Peer led | 1.19 (1.12, 1.27) | <.001 | 1.05 (1.02, 1.09) | 0.01 |
| Facility ART Distribution group | 1.07 (1.01, 1.13) | 0.03 | 1.04 (1.01, 1.08) | 0.01 |
| Fast track | 1.20 (1.13, 1.27) | <.001 | 1.11 (1.07, 1.15) | <.001 |
| Prevention with Positives Package (PwP) |  |  |  |  |
| 0 | ref |  | ref |  |
| 1 | 0.86 (0.85, 0.86) | <.001 | 0.93 (0.92, 0.94) | <.001 |
| 2 | 0.61 (0.60, 0.62) | <.001 | 0.85 (0.84, 0.86) | <.001 |
| 3 or More | 0.59 (0.59, 0.60) | <.001 | 0.85 (0.84, 0.85) | <.001 |
| ART Initiation Year |  |  |  |  |
| 2017 | ref |  | ref |  |
| 2018 | 1.21 (1.20, 1.22) | <.001 | 1.20 (1.19, 1.21) | <.001 |
| 2019 | 1.50 (1.48, 1.52) | <.001 | 1.48 (1.47, 1.49) | <.001 |
| 2020 | 1.68 (1.66, 1.71) | <.001 | 1.83 (1.82, 1.85) | <.001 |
| 2021 | 2.02 (1.99, 2.05) | <.001 | 2.22 (2.20, 2.25) | <.001 |
| 2022 | 2.08 (2.05, 2.12) | <.001 | 2.64 (2.61, 2.68) | <.001 |
| 2023 | 0.63 (0.61, 0.65) | <.001 | 3.51 (3.42, 3.60) | <.001 |
| Covid Pandemic Period |  |  |  |  |
| Non Covid Period | ref |  | ref |  |
| Covid Period | 1.17 (1.16, 1.18) | <.001 | 0.92 (0.91, 0.92) | <.001 |
| Prior Missed Appointment |  |  |  |  |
| No | ref |  | NA ^h.^ | NA |
| Yes | 0.35 (0.34, 0.35) | <.001 |  |  |
| Prior Default Appointment |  |  |  |  |
| No | ref |  | NA | NA |
| Yes | 0.52 (0.51, 0.53) | <.001 |  |  |
|  |  |  |  |  |
| Missed Appointments |  |  |  |  |
| 0 | NA | NA | ref |  |
| 1-to-3 |  |  | 0.93 (0.93, 0.94) | <.001 |
| 4-to-6 |  |  | 0.81 (0.80, 0.82) | <.001 |
| 7+ |  |  | 0.73 (0.72, 0.75) | <.001 |
| Default Appointments |  |  |  |  |
| 0 | NA | NA | ref |  |
| 1-to-3 |  |  | 1.00 (1.00, 1.01) | 0.1 |
| 4-to-6 |  |  | 0.97 (0.96, 0.98) | <.001 |
| 7+ |  |  | 0.95 (0.94, 0.96) | <.001 |
| Duration of Treatment Interruptions |  |  |  |  |
| 0 (No Interruptions) | NA | NA | ref |  |
| Less than 30 Days |  |  | 29.94 (29.63, 30.26) | <.001 |
| 30-to-197 Days |  |  | 19.63 (19.51, 19.75) | <.001 |
| 180-to-364 Days |  |  | 8.15 (8.08, 8.23) | <.001 |
| 365 Days or More |  |  | 2.85 (2.82, 2.88) | <.001 |
|  |  |  |  |  |
|  | ** Concordance = 0.79 (se =0)* |  | ** Concordance = 0.88 (se =0)* |  |

^a^ n=number of observations in the train dataset, i.e. 80% of the entire dataset, Events = number of treatment interruptions.

^b^ CI– Confidence interval; ^c^ ART - antiretroviral therapy; ^d^ Ref – Reference category; ^e^ NRTI - nucleoside analogue reverse transcriptase inhibitor, PI - protease inhibitor; ^f^ INSTI - integrase Strand Transfer Inhibitor; ^g^ NNRTI - non-nucleoside analogue reverse transcriptase inhibitor; ^h^ Not Applicable.

References

1. Ministry of Health, National AIDS & STI Control Program. The Implementation of HIV Differentiated Service Delivery in Kenya Using a Quality Improvement Approach, 2024- Operational Manual. 2024. Available from: https://www.differentiatedservicedelivery.org/wp-content/uploads/Kenya-DSD-manual_June-2024.pdf [accessed Feb 20, 2024]
